# A randomised controlled pilot trial protocol for patient led cognitive gamified training during haemodialysis

**DOI:** 10.1038/s41598-024-79797-y

**Published:** 2024-12-28

**Authors:** Murat Aksoy, Samantha Hunter, Aziz U. R. Asghar, Sunil Bhandari

**Affiliations:** 1 University of York, York, England UK; 2 Hull York Medical School, University of Hull, Hull, UK; 3 School of Agriculture, Policy and Development, University of Reading, Reading, UK; 4 Hull University Teaching Hospitals National Health Service (NHS) Trust, Hull, UK

**Keywords:** Cognitive impairment, Cognitive training, Gamified, Haemodialysis, Intradialytic intervention, Neuroscience, Health care, Medical research, Nephrology

## Abstract

Disruptions in cognitive function have been reported in individuals undergoing haemodialysis and those with chronic kidney disease. This pilot study protocol primarily assesses the feasibility and acceptability of using mobile cognitive gaming apps for patient-led cognitive training during haemodialysis sessions. The protocol consists of three phases: (1) reviewing and evaluating available cognitive gaming apps, (2) conducting focus groups/interviews with people with kidney disease to determine app preferences, and (3) undertaking a quasi-experimental randomised controlled trial to compare cognitive outcomes between a patient-led app intervention group and a standard care control group over four months. Primary outcomes will include changes in cognitive test scores [Montreal Cognitive Assessment (MoCA), Modified Mini-Mental State Exam (3MSE), Rapid Objective Working Memory Assessment (ROWMA)], while secondary outcomes will encompass quality of life measures [Patient-Reported Outcomes Measurement (PROM) Kidney Disease Quality of Life Short Form (KDQoL-SF™) v 1.3, Patient-Reported Outcomes Measurement Information System (PROMIS) Global Health Instrument, European Quality of Life Five Dimension (EQ-5D)]. If demonstrated to be effective, this novel method of utilising gamified cognitive training applications could potentially mitigate cognitive decline and improve the well-being of people receiving haemodialysis without necessitating significant clinical resources. The findings from this research will guide the development of a larger definitive randomised trial in the future.

## Introduction

Cognitive impairment is a significant issue among people with chronic kidney disease (CKD) and people receiving haemodialysis. The CKD-related effect on cognition increases with CKD stage progression and occurs on top of the normal age-related cognitive impairments^1^. Disruptions in cognitive function, characterised by a decline in performance in one or more cognitive domains such as memory, attention, and executive functioning, have been widely reported in the CKD population compared to the general population^2–4^. The prevalence of cognitive deficits in one or more domains ranges from 16–38% depending on the stage of CKD^5–8^. People receiving haemodialysis have anecdotally reported experiencing a ‘brain fog’ with impairments in cognitive function, including memory, thinking, and concentration^9–11^.

Despite the significant impact of cognitive impairment on the quality of life and well-being of people with CKD, there is often a lack of awareness and engagement in addressing this issue^12^. Less than 5% of people with CKD have been evaluated and diagnosed for cognitive impairment, highlighting a substantial gap in recognising and treating cognitive deficits in this population^13–15^. Given the lack of awareness and engagement in addressing cognitive impairment in people with CKD, there is a need for innovative and accessible interventions to mitigate cognitive decline in this population.

Cognitive brain training is a non-pharmacological intervention in which participants undertake a series of cognitive activities with the goal to preserve or enhance their mental abilities, including memory, attention, judgment/decision-making, learning and reasoning^16^. A recent study in people receiving haemodialysis and cognitive training using a classical n-back memory task showed improvements in cognitive and attentional function^17^. The n-back task involves monitoring a sequence of stimuli and identifying when the current stimulus matches the one from n steps earlier in the sequence. It is designed to assess and train working memory and cognitive control by requiring participants to update and maintain relevant information over varying periods of time^18^. However, one problem with such specialised cognitive training tasks is that they can be disengaged to the person as they are repetitive, frustrating and effortful and are typically required to be delivered by a health professional/researcher, thus adding significant resource implications.

Traditional cognitive training tasks often lack engaging elements and require significant effort from the patient, leading to low adherence and high dropout rates^19^. Moreover, the need for delivery by trained professionals limits accessibility and increases resource burdens on healthcare systems^20,21^. In contrast, gamified cognitive training apps offer an accessible, engaging, and self-directed approach to cognitive rehabilitation. By incorporating game design elements such as rewards, challenges, and personalized feedback, these apps can enhance motivation, adherence, and overall user experience. The self-directed nature of gamified apps also reduces the need for direct supervision by healthcare professionals, potentially increasing scalability and cost-effectiveness of cognitive interventions^22^.

A recent approach to cognitive rehabilitation is the use of digital mobile gaming apps delivered via smartphones and tablet devices^23^. Such gamification apps have advantages in terms of adding elements of fun and enjoyment, thereby empowering patients to engage and adhere much more to cognitive rehabilitation tasks. In addition, by tapping into users’ appetite for healthy competition, gamification apps can increase compliance and personal motivation levels. It may also potentially make positive use of the time spent on haemodialysis and have secondary beneficial impacts on carers and family. An investigation by Bento et al. has shown that a digital therapeutic game intervention improved depressive symptoms in elderly patients undergoing haemodialysis treatment^24^. Additionally, McAdams-DeMarco et al. found that playing tablet-based brain games during haemodialysis may prevent cognitive decline^25^. More recently, it has been shown that intradialytic cognitive training interventions can improve functional status of people undertaking haemodialysis^26,27^. There are brain gaming apps available that provide cognitive training (recalling information, attention, problem-solving, and mental agility) and have the potential to be utilised to prevent the decline in cognitive function during haemodialysis^28^. Patient-led cognitive gamified training (CGT) refers to the use of gamified cognitive training apps by patients independently, without direct supervision or guidance from healthcare professionals. This approach empowers patients to engage in cognitive rehabilitation at their own pace and convenience, potentially increasing adherence and effectiveness.

## Research question

The primary research question is: Is it feasible to implement patient-led CGT interventions delivered through smartphone/tablet-based gaming apps during haemodialysis sessions, and what is the potential impact on cognitive function, quality of life, well-being, and functional capacity in people with CKD undergoing haemodialysis?

## Aims and objectives

The overall aim of this trial protocol is to utilise smartphone/tablet-based gaming apps to deliver patient-led CGT during haemodialysis sessions without the direct involvement of health professionals. The specific trial aims are:

- To investigate the effect of patient-led CGT intervention on cognitive decline during haemodialysis.
- To utilise smartphone/tablet-based gaming apps to deliver patient-led CGT during haemodialysis sessions without the direct involvement of health professionals.
- To assess the quality of life, well-being, and functional capacity of people receiving haemodialysis at baseline and the end of the trial.

To achieve these aims, our objectives are to:

- Identify and classify which cognitive apps and games can be used by people with CKD during haemodialysis sessions.
- Develop a CGT intervention for the haemodialysis sessions of people with CKD.
- Develop an understanding of how patient-led CGT during haemodialysis sessions affect:
  - Cognitive decline of people with CKD who undergo haemodialysis therapy, as cognitive function is a key outcome of interest and may be positively influenced by the intervention.
  - Quality of life, well-being and functional capacity of people with CKD who undergo haemodialysis therapy, as these factors are important patient-centred outcomes that may be indirectly impacted by improvements in cognitive function and the engaging nature of the intervention.

## Methods and analysis

### Trial design

**Fig. 1:**
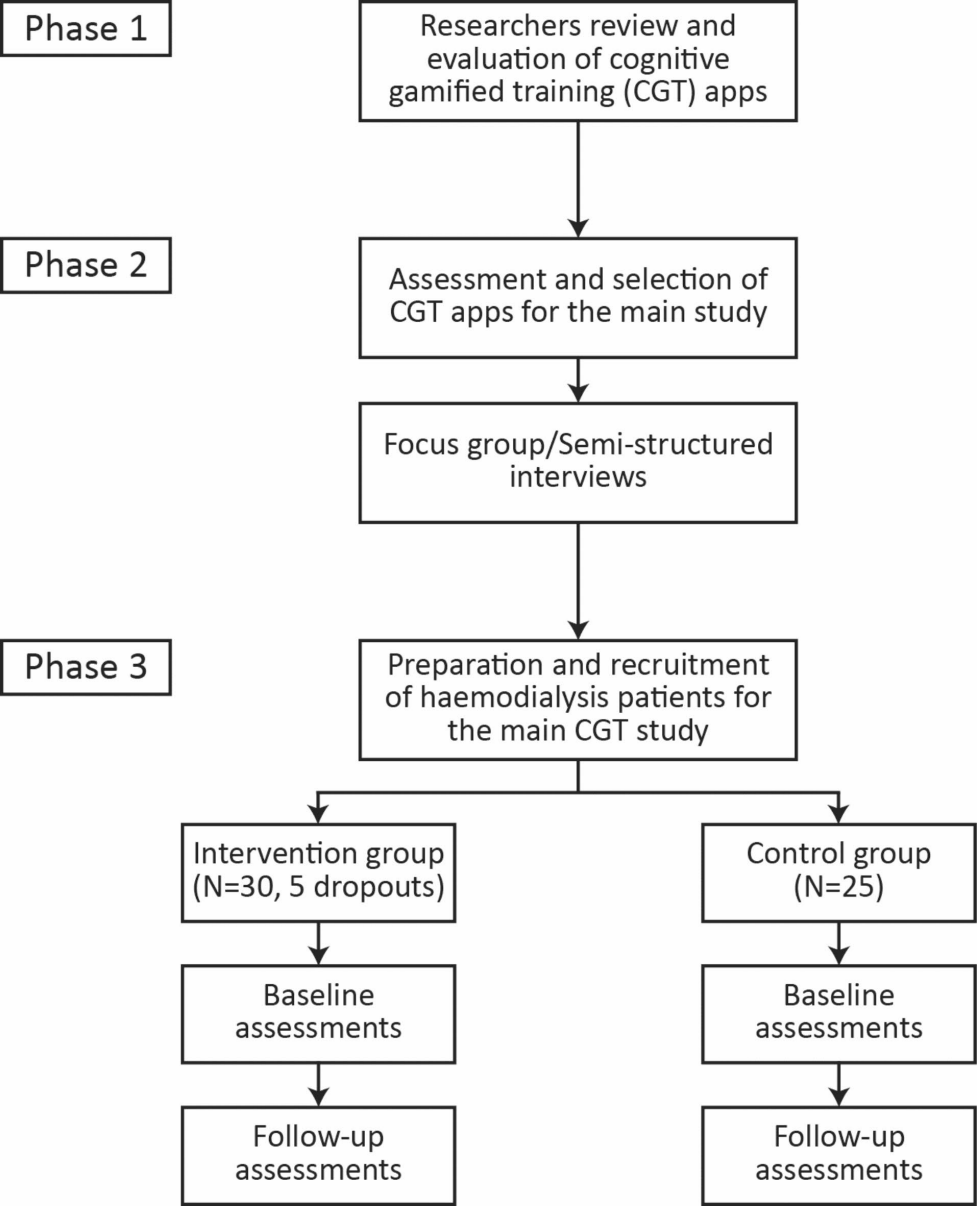
The trial flow diagram.

This trial is a single-centre, two-armed pseudo-randomised controlled pilot trial to assess the feasibility and effectiveness of patient-led CGT intervention for slowing cognitive decline in people receiving haemodialysis. This trial will contain three phases. Phase one will conduct a review and evaluation of CGT apps in the marketplace. The outcome of Phase one is to have a shortlist of CGT apps for the next phase by evaluating cognitive training apps. Phase two will assess and select CGT apps for the intervention phase by conducting semi-structured interviews with the haemodialysis patient focus group involving representatives from the Kidney Patient Associations (KPAs), people with kidney disease, and Kidney Care UK. Phase three will be a pseudo-randomised controlled study in which people receiving haemodialysis will undertake CGT intervention by engaging CGT apps during haemodialysis sessions. The outcome of Phase three will be to have comparisons of baseline and four-month follow-up assessments between the intervention group, which receives CGT apps, and the control group, which only has standard care. The trial flow diagram is presented in Fig. 1.

### Intervention timeline

The CGT intervention will be delivered during the first half of the haemodialysis session, typically within the first 1–2 h, to ensure that patients are alert and able to engage with the cognitive training. Regarding the study procedures:

- Baseline assessments (questionnaires and cognitive tests) will be conducted in the week prior to starting the intervention.
- The intervention will last for four months (16 weeks ± two weeks).
- Patients will be asked to use the cognitive training apps for approximately 30 min during each haemodialysis session, 3 times per week.
- Post-intervention assessments will be conducted in the week following completion of the 16-week intervention.

### Intervention implementation and practical considerations

Participants will be instructed to use their non-fistula/graft arm for interacting with the tablet during CGT to avoid any potential complications. All tablets will have stands to minimise arm movement if required.

Regarding the timing of dialysis shifts, we acknowledge that early morning shifts may pose challenges. To address this, we will ensure that the CGT apps are designed with adjustable brightness and volume settings to accommodate patient comfort at different times of day.

The involvement of dialysis staff will be minimal to ensure the intervention remains patient-led. Staff responsibilities will include:

- Initial setup of tablets (performed by the research team).
- Providing tablets to patients at the start of their session.
- Basic troubleshooting if needed (e.g., restarting an app).
- Collecting tablets at the end of the session.

Staff will receive a brief training session on these responsibilities. We estimate this will require no more than 5–10 min of staff time per patient per session. The research team will be available to address any technical issues that arise.

### Trial population and recruitment

The investigators will collaborate with local representatives of KPAs and people within the kidney service to recruit the participants who are currently receiving haemodialysis therapy at the Hull University Teaching Hospitals NHS Trust kidney service. Healthcare professionals at the kidney unit will discuss study participation with the patients before obtaining formal informed consent.

The investigators will give potential participants a letter and the trial participant information sheet. Consent for participation in the trial will be collected via a formal Informed Consent Form authored by the investigators; the consent form will check candidates suitability against both inclusion and exclusion criteria (fit recruitment profile/ confirmation that situations have not changed since recruitment), and outline the specifics of the trial, what activity is included in the intervention sessions (methodology outline) and how both feedback and personal data will be collected and processed. All inclusion and exclusion criteria are given in Table 1. For Phase two, the focus group/semi-structured interview sessions will be conducted with people who meet the same inclusion and exclusion criteria as the main trial participants (Table 1), ensuring consistency in the target population.

**Table 1:** Inclusion and exclusion criteria of the proposed trial.

Inclusion criteria	Exclusion criteria
18 years or older	Less than 18 years of age
regular haemodialysis therapy for at least three months	Haemodialysis for less than three months
	Planned kidney transplant within the intervention period
	Active malignancy
	Inability to complete study assessments from the clinical perspective
	Inability to complete the interventions from the clinical perspective
	Inability to use tablet and the CGT app Inability to consent to study

As this is a pilot study, the sample size is determined by the number of eligible patients in the participating haemodialysis unit. While formal age-matching between the intervention and control groups is not feasible due to the small sample size, we will strive for balance in baseline characteristics through randomisation. The primary aim of this pilot study is to assess feasibility and gather preliminary data to inform a future, larger randomised controlled trial.

### Participant withdrawal

Participants have the right to withdraw from treatment and/or the trial at any time. Participants will remain in the trial for follow-up unless they request to fully withdraw. All data up to the point of withdrawal will be stored, after withdrawal participant’s data will be destroyed and will not be included in the final dataset.

In the event of participant withdrawal, the Investigator(s) will promptly explain to the participant that their involvement in the trial will discontinue and explain why.

For Phase two, the focus group/semi-structured interview sessions will be conducted with people who are currently receiving haemodialysis therapy. After receiving feedback from the focus groups/semi-structured interviews, a finalist of CGT apps will be determined to be used during haemodialysis sessions.

Fifty-five people attending haemodialysis therapy will be recruited for Phase three. The intervention arm includes 30 patients to account for up to a 20% potential dropout and ensure at least 25 can complete the study. The control arm will have 25 haemodialysis patients with usual care. All participants will complete assessments at baseline and at approximately four months (± two weeks).

### Randomisation

Participants will be assigned to either the intervention or control group based on their pre-existing haemodialysis schedule. The dialysis unit operates two main haemodialysis therapy groups: group 1 (Monday, Wednesday, Friday) and group 2 (Tuesday, Thursday, Saturday). Each main group is further divided into three sub-sessions based on the time of day: morning, afternoon, and evening. This results in a total of six possible haemodialysis therapy routes:

- Route 1: Monday, Wednesday, Friday; Morning.
- Route 2: Monday, Wednesday, Friday; Afternoon.
- Route 3: Monday, Wednesday, Friday; Evening.
- Route 4: Tuesday, Thursday, Saturday; Morning.
- Route 5: Tuesday, Thursday, Saturday; Afternoon.
- Route 6: Tuesday, Thursday, Saturday; Evening.

In Phase 3 of this pseudo-randomised controlled study with a parallel design, participants in each of the six routes will be allocated to either the intervention or control arm. To minimise potential bias and contamination between groups, all participants in a given route (e.g., Monday, Wednesday, Friday; Morning/Afternoon/Evening) will be assigned to the same study arm. This approach ensures that intervention and control group participants will not have overlapping haemodialysis schedules, reducing the likelihood of communication and influencing between groups.

The allocation of each route to either the intervention or control arm will be determined by the research team. This pseudo-randomisation method, while not truly random at the individual level, offers a pragmatic approach to group allocation given the scheduling constraints of the haemodialysis unit and the need to minimize potential bias between study arms.

### Trial treatment, data collection and analysis

#### Phase one: researcher review and evaluation of CGT apps in the marketplace

- Collect and evaluate brain training apps from Google Play and the App Store. For the purpose of this study, brain training apps are operationally defined as mobile applications that provide non-invasive, readily accessible, and self-directed gamified cognitive tasks targeting various cognitive domains, including memory, attention, problem-solving, and processing speed^29^. These apps are designed to actively engage users with game-like exercises during dialysis sessions, encouraging them to independently practice their cognitive functions. The selection process will involve collecting and evaluating brain training apps available on Google Play and the App Store based on their adherence to this operational definition and their suitability for use in the haemodialysis setting at the unit.
- Shortlist apps based on their usefulness for cognitive training. While gaming apps may offer an accessible and engaging approach during haemodialysis sessions, it is important to note that their validity compared to standard cognitive assessments administered by trained professionals has not been established specifically in the haemodialysis population. Therefore, we will use validated cognitive assessment tools (MoCA, 3MSE) as our primary outcome measures, with the gaming apps serving primarily as an engagement tool during treatment sessions rather than a cognitive assessment instrument.
- All data collection and analysis in Phase one will be conducted by the investigators, without patient involvement.

These areas will guide the development of specific questions, which will be refined based on input from the research team and piloting with a small group of patients. This approach will ensure a comprehensive assessment of app usefulness while allowing flexibility to explore emerging themes during discussions. Focus groups/semi-structured interviews will be conducted by trained members of the research team, including study investigators and research assistants.

#### Phase two: assessment and selection of CGT apps for the main trial in conjunction with the kidney patient groups

- Conduct focus groups/semi-structured interviews with haemodialysis patients to assess the usefulness of cognitive training apps during dialysis sessions.
- Collaborate with KPAs and Kidney Care UK representatives.
- The interview structure will be based on the Technology Acceptance Model (TAM^30^), which is widely used to evaluate user acceptance of new technologies. Key areas of inquiry will include:
  - Perceived ease of use (e.g., How easy or difficult was it to use the app during dialysis? ).
  - Perceived usefulness (e.g., In what ways might this app be beneficial during dialysis sessions? ).
  - Attitude towards using (e.g., What are your overall thoughts on using this app during dialysis? ).
  - Behavioural intention to use (e.g., How likely would you be to use this app regularly during future sessions? ).

#### Phase three: undertake CGT apps intervention in people receiving haemodialysis

- Perform baseline assessments: Montreal Cognitive Assessment (MoCA), Modified Mini-Mental State Exam (3MSE), Rapid Objective Working Memory Assessment (ROWMA), Patient-Reported Outcomes Measurement (PROM) Kidney Disease Quality of Life Short Form (KDQoL-SF^™^) v 1.3, Patient-Reported Outcomes Measurement Information System (PROMIS) Global Health Instrument, European Quality of Life Five Dimension (EQ-5D) for cognitive function, quality of life, and well-being.
- The combination of cognitive assessments (MoCA, 3MSE, ROWMA) provides an evaluation of cognitive function, ranging from screening for mild impairment to specific assessment of working memory. For quality of life, the use of both disease-specific (KDQoL-SF™) and generic (EQ-5D, PROMIS) measures allows for an understanding of the intervention’s effects on QoL. This multi-faceted approach enhances the validity and reliability of our findings, capturing both general and kidney disease-specific aspects of cognitive function and quality of life that may be impacted by the intervention.
- In the intervention group, patients will use the selected CGT apps on tablet devices during haemodialysis sessions for a minimum of 30 min per session, 3 times per week, over the 4-month intervention period. The research team members will provide initial training on app use and will be available to assist patients as needed throughout the intervention. Regular check-ins with patients will be conducted to discuss their experiences and address any barriers to app use.
- In the control group, provide standard care.
- After four months, repeat assessments (MoCA, 3MSE, ROWMA, KDQoL-SF^™^, PROMIS, EQ-5D) and compare results between intervention and control groups.

Data collection and analysis will be conducted by investigators and trained study staff. Due to the nature of the intervention, blinding is not possible in this study. Participants will be aware of whether they are receiving the cognitive training intervention or standard care. Dialysis staff will also be aware of which patients are using the cognitive training apps during dialysis sessions. Additionally, outcome assessors cannot be blinded in this study design. The visible presence of tablets and app usage during dialysis sessions for the intervention group makes it impossible to conceal group allocation from the assessors.

We acknowledge that the lack of blinding at all levels is a limitation of our study design. This is a common challenge in studies of behavioural interventions in clinical settings, particularly those involving technology use during treatment. To mitigate potential bias despite the lack of blinding:

1. We will use standardized assessment protocols to ensure consistent data collection across all participants.
2. Where possible, we will rely on objective measures (e.g., app usage data, standardized cognitive tests) to minimize subjective influences.
3. Multiple assessors will be used to reduce individual bias.

We will thoroughly discuss this limitation and its potential implications in our final report, considering how it might impact the interpretation of our results.

### Outcomes

The primary outcome will be a change in cognitive test results (MoCA, 3MSE and ROWMA) of the intervention group at approximately four-month (± two weeks) follow-up assessments compared to the baseline assessments. The secondary outcomes will be the change in the behavioural test scores (PROM KDQoL-SF™ v1.3, PROMIS Global Health Instrument and EQ-5D questionnaires) for the CGT intervention group compared to the control group. The following validated instruments will be used to assess cognitive function, quality of life, and general health and well-being:

- MoCA: A sensitive screening tool for mild cognitive impairment, assessing various cognitive domains including attention, concentration, executive functions, memory, language, visuoconstructional skills, conceptual thinking, calculations, and orientation^31^.
- 3MSE: An expanded version of the MMSE that provides a comprehensive and sensitive measure of global cognitive function^32^.
- ROWMA: A brief, computerised test specifically designed to assess working memory, which is often impaired in patients with kidney disease^33^.
- KDQoL-SF™: A kidney disease-specific measure of health-related quality of life that includes generic core plus disease-specific domains such as symptom/problems, effects of kidney disease on daily life, burden of kidney disease, cognitive function, and dialysis staff encouragement^34^.
- PROMIS Global Health Instrument: A brief, generic self-report measure of health-related quality of life that assesses physical, mental, and social health^35^.
- European Quality of Life 5-D (EQ-5D): A standardized instrument for measuring generic health status, providing a simple descriptive profile and a single index value for health status^36^.

Table 2 provides a summary of these assessment tools, including their functions, score ranges, administration details, and other relevant information.

**Table 2:** Summary of assessment tools.

Assessment tool	Function/rationale	Score range and interpretation*	Administration timing	Time to complete	Other details
Montreal Cognitive Assessment (MoCA)	Screens for mild cognitive impairment	0–30; ≥26 normal	Baseline and 4 months	10–15 min	Assesses multiple cognitive domains
Modified Mini-Mental State Exam (3MSE)	Comprehensive measure of global cognitive function	0-100; higher scores indicate better cognitive function	Baseline and 4 months	10–15 min	More sensitive than standard MMSE
Rapid Objective Working Memory Assessment (ROWMA)	Assesses working memory	Scores vary; higher scores indicate better working memory function	Baseline and 4 months	5–10 min	Computerised test
Kidney Disease Quality of Life Short Form (KDQoL-SF™)	Kidney disease-specific quality of life measure	0-100 for each subscale; higher scores indicate better quality of life	Baseline and 4 months	10–15 min	Includes generic and kidney disease-specific components
PROMIS Global Health Instrument	Brief measure of general health-related quality of life	T-score metric (mean = 50, SD = 10); higher scores indicate better health	Baseline and 4 months	5–10 min	Assesses physical, mental, and social health
European Quality of Life 5-D (EQ-5D)	Generic measure of health status	Index value 0–1; higher scores indicate better health status	Baseline and 4 months	5–10 min	Provides health utility scores for economic evaluations

*Score ranges and interpretations are based on general population normative data unless otherwise specified. Specific cutoff values and interpretations for the haemodialysis population may differ and are currently not well established. This highlights the exploratory nature of using these assessment tools in our specific.

Examining and comparing the cognitive and behavioural test scores of the intervention and control groups at the four-month assessments will provide insight to expand the scope and area of CGT intervention to other haemodialysis units. Feasibility outcomes will include patient recruitment and retention rates, adherence to the intervention (app usage frequency and duration), and the occurrence of any technical issues or adverse events. Acceptability will be assessed through semi-structured interviews with a subset of intervention group participants, exploring their experiences, satisfaction, and perceived benefits and challenges of using the CGT apps. If the feasibility and acceptability of CGT intervention are demonstrated, we will apply for further funding so that the CGT intervention can be delivered to more people receiving haemodialysis.

### Statistical analysis

Given the pilot nature of this study, our sample size is not based on formal power calculations. Instead, it is pragmatically determined by the number of eligible patients in our dialysis unit. The results will be used to estimate effect sizes and inform sample size calculations for a future, full-scale randomised controlled trial.

In Phase three, data will be analysed to test the effect of CGT intervention on the scores of baseline and follow-up assessments. All data analyses will be conducted in IBM SPSS Statistics for Windows, version 27.0 (or higher) software. Data will be directly entered into Microsoft Office Excel spreadsheets (version 2202) by investigators and study staff using all standard data management procedures. Descriptive statistics will be calculated according to the normality assumption, with means and standard deviations reported for normally distributed data, and medians and interquartile ranges reported for non-normally distributed data. Within-group comparisons between baseline and follow-up assessments for primary and secondary measurements will be analysed using repeated-measures analysis of variance (ANOVA). In contrast, independent ANOVA will be used for between-group comparisons. For additional explorative analyses, correlation and regression models can be used to analyse the effects of other factors (e.g. age, gender) on the cognitive decline between the baseline and follow-up assessments.

In addition to the primary and secondary outcome analyses, a sub-group analysis will be performed to explore the potential effects of the CGT intervention based on participants’ baseline memory function and executive function. Participants will be stratified into subgroups based on their performance on the cognitive test scores, and the intervention effects will be compared between these subgroups. This exploratory analysis may help to identify which patients are most likely to benefit from the CGT intervention and guide future research in this area.

No formal power calculation was conducted because this pilot study focuses on evaluating the feasibility and acceptability of using CGT apps in individuals undergoing haemodialysis. The primary goal of this pilot trial is to collect initial data and assess the study procedures rather than testing a specific hypothesis with a predetermined level of statistical power. The results from this pilot study will guide the design and sample size calculation for a future, larger scale definitive randomised controlled trial.

While our sample size limits a detailed analysis of the impact of different dialysis schedules, we will collect data on each participant’s schedule (Monday-Wednesday-Friday or Tuesday-Thursday-Saturday, and shift time). We will conduct exploratory analyses to examine potential associations between schedule and outcomes. This information will be valuable for designing a larger future trial where schedule effects could be more robustly analysed.

### Regulatory aspects

The RCT will be conducted according to the standards of the ICH-Good Clinical Practice (GCP) and the Research Governance Framework for Health and Social Care. Pharmaco-vigilance reporting will comply with the Medicines for Human Use (Clinical Trials) Regulations 2004 and Amended Regulations 2006. Written informed consent will be provided by all patients prior to randomisation and any study-related procedures.

### Ethical considerations

This trial has received ethical approval (IRAS: 312024). Ethical approval for Patient-Led Cognitive Gamified Training in Haemodialysis (PACE) was granted by the National Research Ethics Service Committee London - Harrow (22/LO/0538) on 21 September 2022. This study will be conducted in accordance with all relevant regulations and guidelines, including the Declaration of Helsinki. The privacy and personal information of participants will be protected. The methodologies chosen (focus groups/semi-structured interviews, questionnaires and CGT apps) are unlikely to pose risks to participants.

## Discussion

This pilot protocol was written because high quality studies on the effects gaming apps on cognitive function in people receiving haemodialysis are lacking. CKD and haemodialysis treatment can lead to cerebral hypoperfusion, inflammation, and oxidative stress, which may contribute to cognitive decline^1,9,37,38^. Cognitive gaming may help counteract these effects by promoting neuroplasticity, increasing cerebral blood flow, and potentially reducing inflammation^39–41^. By providing regular cognitive stimulation and engagement during haemodialysis sessions, games may help maintain and potentially improve cognitive reserve, which is particularly important in this vulnerable population. The design of our intervention, which combines cognitive stimulation with the convenience of intradialytic delivery, aims to leverage these potential benefits while addressing the practical constraints of haemodialysis treatment.

In addition, published studies have failed to provide sufficient data. The results of PACE will provide evidence on whether using gaming apps is feasible and useful and potentially beneficial to people receiving haemodialysis and provide valuable information to the design of a definitive randomised controlled trial. It is therefore hoped that this pivotal study can provide new findings to allow future consideration of a large randomised controlled trial. Recent studies have shown promising results for the use of cognitive and physical training interventions in haemodialysis patients. These findings suggest that non-pharmacological, bimodal interventions combining cognitive training and physical exercise may be an effective approach to mitigate cognitive decline and improve overall well-being in the haemodialysis population. Our protocol aims to systematically evaluate this hypothesis through a carefully designed pilot study. 

## Data Availability

The datasets generated and/or analysed during the current study are not publicly available due to this being a protocol paper with no datasets generated and/or analysed yet but will be available from the corresponding author on reasonable request following study completion.
